# Effect of Polysaccharides From *Enteromorpha intestinalis* on Intestinal Function in Sprague Dawley Rats

**DOI:** 10.3389/fphar.2021.796734

**Published:** 2022-01-28

**Authors:** Xia Li, Miao Zhang, Hock Eng Khoo, Tiemin Jiang, Yuan Guan, Peijun Li

**Affiliations:** Guangxi Key Laboratory of Electrochemical and Magnetochemical Functional Materials, College of Chemistry and Bioengineering, Guilin University of Technology, Guilin, China

**Keywords:** seaweed, proximate analysis, intestinal function, plant metabolites, probiotics

## Abstract

This study aims to determine the effect of polysaccharides extracted from *Enteromorpha intestinalis* (EI) on the intestinal function of Sprague Dawley (SD) rats. The polysaccharides were extracted from the green alga using water and alkaline solution, where these extracts were named WPEI and APEI, respectively. The dried powder of EI was labeled as DPEI. Proximate compositions, minerals, and amino acids of the DPEI, WPEI, and APEI were determined. The growth-promoting effect of the polysaccharides on selected intestinal microflora was determined based on the plate count method. In contrast, the *in vivo* effect of DPEI and its polysaccharides on the intestinal function of the SD rats was determined. These rats were fed with 1% WPEI, APEI, and DPEI. The result showed that APEI had lower total sugars and total proteins content than the WPEI. WPEI did not contain arabinose. The WPEI and APEI also had a better ability to promote microbial growth than the DPEI. The *in vivo* study showed that WPEI improved intestinal peristalsis and other intestinal functions compared with the other rat groups. The average final body weight of the experimental rats treated with DPEI was also lower than the other groups. The pH value of the feces of all treated rats was lower than the control rats, and the moisture content of the fecal samples of these experimental groups was higher than the control group. Also, the intestinal activated carbon propulsion of the WPEI, APEI, and DPEI fed rats increased. Among the short-chain fatty acids content determined in the fecal samples, the propionic acid content of the WPEI group was significantly highest. Therefore, WPEI had the best effect in improving intestinal digestion.

## Introduction

Intestinal microflora is crucial in maintaining gut health because the gastrointestinal tract is related to many diseases. These diseases are closely linked to the gut microbiota. As prebiotics are food for the microflora, the substances play a role in improving intestinal health. Studies have found that various plant polysaccharides have improved intestinal health. Alga is a rich source of natural polysaccharides. Among the algae, *Enteromorpha (Ulva) intestinalis* (EI) has a high amount of polysaccharides. It is one of the green lavers traditionally used as food in Japan, Korea, and China. Literature shows that EI biomass can be used to adsorb contaminants (El-Naggar et al., 2021). The EI extracts possessed antioxidative, anti-inflammatory, and anti-diabetic effects besides the polysaccharides content (Pradhan et al., 2021). The molecular structures and bioactivities of EI polysaccharides have also been determined. The biological activities of polysaccharides from *Enteromorpha* are antioxidant (Shi et al., 2017), hypoglycemia (Yuan et al., 2019), hypolipidemia (Teng et al., 2013), immunomodulatory (Wei et al., 2014), and prebiotic activity (Praveen et al., 2019).

There is a relationship between the biological activity of plant polysaccharides and the extraction method. The structure and bioactivity of polysaccharides extracted using different extraction methods could be different. A previous study found that the seaweed polysaccharide has a growth-promoting effect on beneficial intestinal bacteria, such as *L. plantarum* NCIM 2083. It inhibited the growth of pathogenic intestinal bacteria *Salmonella typhimurium* MTCC 3224 (Praveen et al., 2019). *In vitro* fermentation of the polysaccharides from *E. prolifera* promoted the proliferation of the probiotic strains (*Lactobacillus* and *Bifidobacterium*) (Kong et al., 2016). However, there is still a lack of scientific study to evaluate the effects of *E. prolifera* on probiotics and intestinal function. Studies have shown that the intestinal microflora, fecal water content, intestinal pH, and intestinal microflora metabolite content were the main factors affecting intestinal health (Huang et al., 2014; Makki et al., 2018). Lentinan and *Dendrobium candidum* polysaccharides also regulated the intestinal function, such as participating in the immune process of the human body by acting on the intestinal mucosa, protecting the integrity of the intestinal barrier structure and function, regulating the composition of the intestinal flora, and stimulating the intestine endocrine (Groschwitz and Hogan, 2009; Xie et al., 2016; Jiang et al., 2019).

Plant polysaccharides are prebiotics because they are undigestible in the digestive tract, but they can be metabolized into other compounds by the intestinal microflora. The prebiotics regulate and promote the microflora growth in the large intestine and inhibit the proliferation of pathogenic bacteria (Kong et al., 2009). There is also no recent study reporting the differences in prebiotic activities between water and alkaline extracts of EI polysaccharides. Therefore, this study aims to determine the effects of EI polysaccharides on the growth of selected probiotic strains. The prebiotic effect of the polysaccharides on improving the intestinal function of rats was also studied. It provided a theoretical basis for the potential development and utilization of EI polysaccharides.

## Materials and Methods

### Chemicals and Samples

EI samples were collected in the coaster area of Zhejiang, China. *Lactobacillus brevis*, *L. plantarum*, *L. bulgaricus*, and *Streptococcus thermophilus* were obtained from the Guangdong Microbial Strain Preservation Center. Isopropanol purchased was of chromatographic grade, and all other chemicals and reagents were of analytical grade (Xilong Chemical Co., Ltd., Guangdong, China).

### Sample Preparation

The freeze-dried powder of EI (DPEI) was prepared by freeze-drying the fresh EI sample, followed by pulverizing and sieving at 40 mesh. The WPEI sample (water extract of EI polysaccharides) was prepared by extracting the DPEI for 3 h in a water bath at 90°C (1:40, w/v). The APEI sample (alkaline extract of EI polysaccharides) was obtained by extracting the freeze-dried powder with 2% sodium hydroxide and then incubated in a 75°C water bath for 2 h (1:40, w/v). All extract solutions were filtered, concentrated, and precipitated with 80% ethanol. The precipitates were finally centrifuged at 4,000 rpm for 5 min and freeze-dried.

### Proximate and Chemical Analyses

The measurements of the proximate compositions, minerals, and amino acids content of EI samples were performed in accordance with the methods reported in the literature (Alcântara et al., 2017). Crude protein content was measured using the Coomassie brilliant blue method. Crude polysaccharide content was determined based on the phenol-sulfuric acid reaction system. Crude fiber content was determined using the gravimetric method, which was in accordance with China National Food Standard (2003) GB/T 5009.10-2003. The uronic acid content was measured using the m-hydroxydiphenyl method with D-galacturonic acid as standard. The determination of sulfate content was performed based on the spectrophotometric method (Jiao et al., 2009; Wang et al., 2018). The monosaccharide compositions of the EI samples were determined based on the gas chromatographic method described by Jiao et al. (2009) after hydrolysis with 2 M trifluoroacetic acid.

### Microbial Growth Assays

The effects of EI polysaccharides on the growth of *L. brevis*, *L. plantarum*, *L. bulgaricus*, and *S. thermophilus* were determined using the plate colony counting method (Kong et al., 2016; Sun et al., 2021). In brief, the probiotic strains (1.0 × 10^–5^) were cultured at 37°C for 48 h in MRS media containing the polysaccharide samples. After 48 h, microbial growth was observed, and the CFU of each probiotic strain was obtained. A total of five groups were included in this microbial assay. They were MRS medium with 2% glucose (control group, A), MRS medium with 2% WPEI (B), MRS medium with 2% APEI (C), MRS medium with 1% glucose and 1% WPEI (A + B), and MRS medium with 1% glucose and 1% APEI (A + C). The results were expressed as log_10_ CFU/mL.

### Animal Experimentation

Twenty-four male Sprague Dawley rats weighing 250 ± 30 g were obtained from Slack Jingda Experimental Animal Co, Ltd. (Hunan, China). All rats used in this study were caged according to the standards of animal management regulations (Hu et al., 2012). Ethical approval (number: 2021040917) was obtained from the Animal Care and Use Committee (ACUC), Guangxi Institute of Botany, Chinese Academy of Sciences (Guilin, China). Animal handling was performed in accordance with the institutional ACUC guidelines.

The experimental rats were acclimatized for 1 week with free access to filtered tap water and rat chow. The rat chow was purchased from a local animal feed supplier, and the chemical composition of the rat chow (basic diet) is shown in Table 1. The ambient temperature was maintained at 25 ± 3°C and relative humidity of 50 ± 5% with a 12 h light/dark cycle. Before the experiment, the rats were randomly divided into four groups (*n* = 6). Each rat was caged individually. They were control group fed with only a basic diet. The DPEI group was fed a basic diet containing 1.0% DPEI, the WPEI group was fed a basic diet containing 1.0% WPEI, and the AEPI group was fed a basic diet containing 1.0% APEI. The dose was selected according to the dosage reported in the literature (Tair et al., 2018). All rats were fed with the experimental diet for 40 days before being sacrificed.

**TABLE 1:** Composition of the basic feed.

Components	Proportion (%)
Corn starch	51.2
Casein (protein ≥85%)	14
Gelatinized corn starch	15.5
Sucrose	10.9
Soybean oil	4
Lard	1.5
Choline bitartrate	0.07
Mineral mix^a^	2.8
Vitamin mix^b^	0.03

a Mineral mix: sodium chloride, magnesium chloride, lithium chloride, sodium fluoride, potassium sulfate, chromium (III) potassium sulfate dodecahydrate, calcium carbonate, manganese carbonate, zinc carbonate hydroxide, copper (II) carbonate hydroxide, potassium dihydrogen phosphate, potassium iodate, ammonium metavanadate, boric acid, selenium dioxide, ammonium paramolybdate tetrahydrate, sodium metasilicate nonahydrate, potassium isocitrate, and iron citrate.

b Vitamin mix: vitamin A, vitamin B1, vitamin B2, vitamin B3, vitamin B6, vitamin B9, vitamin B12, vitamin D3, vitamin E, vitamin K, biotin, and calcium pantothenate.

### Anthropometric Measurements and *In Vivo* Biochemical Analyses

Body weights (BWs) and food intakes of all experimental rats were measured at baseline and 4-day intervals until the end of the study. The rats were weighed and recorded before the feeding. The food efficiency ratio was calculated as follows:

$$\text{Food}\;\text{efficiency}\;\text{ratio}=\frac{M_{1}}{M}$$ (1)

where *M*_1_ is the average daily weight gain (g) and *M* is the average daily food intake (g).

On day 25, the fresh feces of the rats were collected to determine moisture content, pH values, and short-chain fatty acids (SCFAs). At the end of the experiment, all rats were sacrificed. The stomach, spleen, kidney, thymus, and liver of all rats were collected. The weights of these visceral organs were then recorded.

### Determination of Activated Carbon Propulsion

The percentages of activated carbon propulsion of the experimental rats were determined according to the method described by Qian et al. (2018). During the last day of the study, all rats were fasted before performing the activated carbon propulsion test. After the 8 h fasting, the rats were forced-fed with 2 ml of 1% EI or the polysaccharides containing 2% medical-grade activated carbon by oral gavage. An hour later, all experimental rats were sacrificed and dissected. The abdominal cavity was dissected, and the small intestine was removed. The length of the small intestine and propulsive length were then recorded. The activated carbon propulsion (P) of the experimental rats was calculated according to the following equation:

$$\text{P}\left(\text{\%}\right)=\frac{t_{1}}{t}\times 100,$$ (2)

where *t*_1_ is the length of gastrointestinal transit (activated carbon propulsive distance, mm) and *t* is the length of small the intestine (mm).

### Determination of Short-Chain Fatty Acids Content of the Feces

The changes in the SCFAs content of the rat feces were determined. The rat feces were freshly collected and added with isopropanol at a ratio of 1:5 (w/v). The mixture was sonicated for 1 h and centrifuged at 4,000 rpm for 5 min. The supernatant was collected and then added with a half volume of anhydrous sodium sulfate, homogenized, and placed at −20°C for 12 h. Finally, the supernatant was filtered through a 0.22 µm syringe filter before performing gas chromatographic analysis.

An Agilent 6890N Network Gas Chromatographic System was used to quantify the SCFAs. The GC-FID conditions for the SCFA separation were as follows: the temperature was programmed as the initial temperature of 85°C, maintained for 2 min; increased to 200°C at 15°C per min; and run for 3 min at 230°C. The temperature of the flame ionization detector was set at 250°C, carrier gas of high purity helium (99.999%), air carrier flow of 1.5 ml/min, injection temperature of 200°C, and injection of 2 µl. The SCFAs content was calculated as follows:

$$\text{SCFAs}\;\text{content}\;\left(\text{µg}/\text{g}\right)=\frac{C_{0}\times 5M}{M\times \left(1-W\right)},$$ (3)

where *C*_0_ is the SCFA concentration in the samples (µg/ml), *M* is the weight of rat feces (g), and W is the moisture content of the rat feces (ml).

### Statistical Analyses

All data were expressed as mean ± standard deviation, except for the chemical compositions of EI and its polysaccharides. The differences between the two groups were analyzed by independent samples *t*-test and analysis of variance coupled with posthoc LSD test using SPSS Statistics 17.0 software. Significant differences between the two groups were set at *p* < 0.05.

## Results

### Proximate Composition and Chemicals Content

The proximate composition of DPEI is shown in Table 2. The crude polysaccharide content of the DPEI was the highest, followed by ash, crude fiber, moisture, and crude protein content. Besides the macronutrients, the DPEI contained aluminum, iron, and silicon as the major minerals, where the iron concentration was 4,300 mg/kg. The DPEI also had 8.41% total amino acids. The non-essential amino acids content of DPEI was 2.5 times higher than the essential amino acids.

**TABLE 2:** Proximate composition, minerals, and amino acids content of DPEI.

	Nutrients	Content (%)	Literature (%)^a^
Proximate composition	Moisture	6.54	10.74–12.15
	Ash	19.10	11.75–17.35
	Crude protein	4.86	4.67–13.42
	Crude fat	ND	0.02–1.31
	Crude carbohydrate	ND	58.03–70.64
	Polysaccharide	40.22	ND
	Crude fiber	7.64	ND
	Chlorophylls	ND	4.07–5.89
Minerals	Aluminium (Al)	0.97	ND
	Iron (Fe)	0.43	0.22–0.34
	Silicon (Si)	0.26	ND
	Iodine (I)	0.04	ND
	Manganese (Mn)	0.08	0.08–0.09
	Zinc (Zn)	0.05	∼0.01
	Copper (Cu)	0.05	0.01–0.02
	Bromide (Br)	0.02	ND
Amino acids	Threonine (Thr)^b^	0.92	ND
	Valine (Val)^b^	0.96	
	Asparagine (Asn)	1.61	
	Glutamate (Glu)	0.97	
	Glycine (Gly)	1.18	
	Alanine (Ala)	1.73	
	Lysine (Lys)^b^	0.48	
	Total essential amino acids	2.36	
	Total non-essential amino acids	6.05	
	Total amino acids	8.41	

a Fresh EI, sample: Metin and Baygar (2018).

b Essential amino acids. Only selected amino acids are reported in Table 2. The chemical compositions of DPEI were presented as means of two replicates; DPEI contained phytochemicals such as chlorophyll, phenolic compounds, and terpenoids, which accounted for over 10% DPEI. ND: not determined.

The chemical compositions of WPEI and DPEI are shown in Table 3. The results showed that WPEI had higher total sugars, total proteins, and sulfate content than APEI. The total sugars content of WPEI was two times higher than the amount determined in APEI. The total protein content of WPEI was also more than seven times higher than the amount found in APEI. Among the monosaccharides determined, Rha, Xyl, Glc, and GlcA content of WPEI was higher than the APEI. It shows that these monosaccharides are highly soluble in water.

**TABLE 3:** Chemical compositions of EI polysaccharides.

Composition (%)	WPEI	APEI
Total sugars	33.30	15.43
Total proteins	7.55	0.98
Sulfate	7.50	5.70
Monosaccharides		
Rhamnose (Rha)	58.00	15.93
Xylose (Xyl)	13.54	5.29
Arabinose (Ara)	—	10.93
Mannose (Man)	2.26	18.04
Galactose (Gal)	2.27	13.09
Glucose (Glc)	20.50	11.96
Uronic acid	2.70	1.10

The chemical compositions (%) of EI polysaccharides were presented as means of two replicates.

### Microbial Growth Assay

**FIGURE 1:**
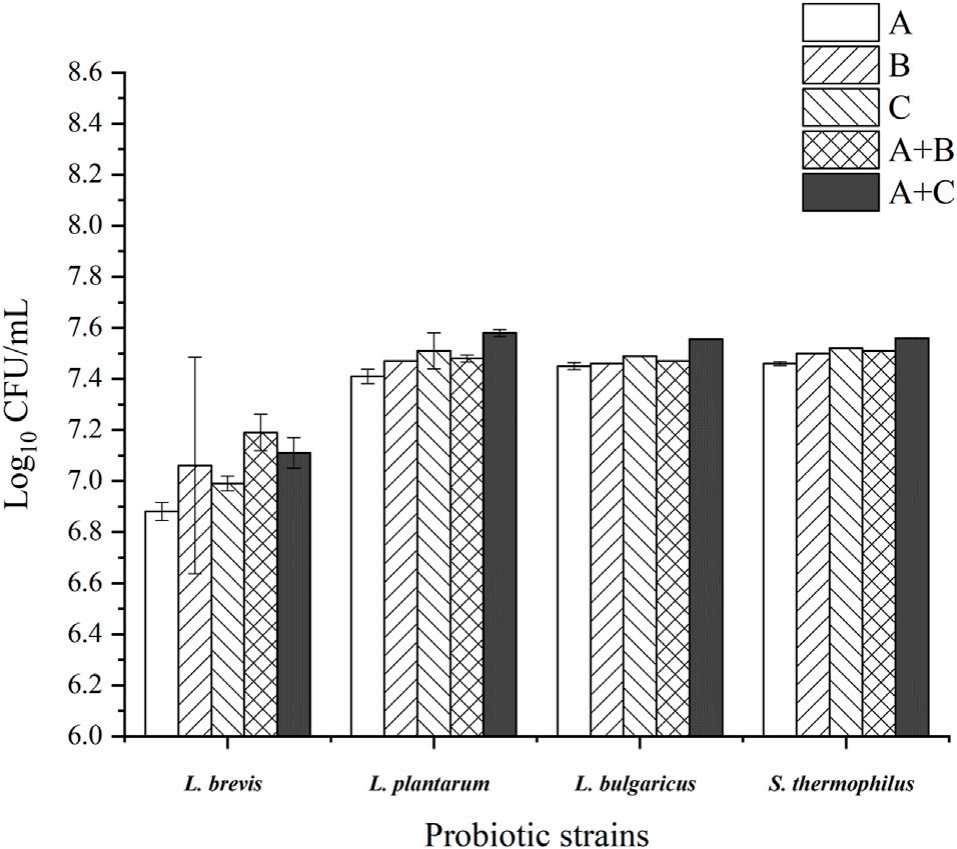
Log_10_CFU of the probiotic strains after 48 h of incubation with the EI polysaccharides. The data were presented as mean ± standard deviation of three replicates. **(A)**: control group; **(B)**: APEI group; **(C)**: WPEI group; A + B: 1% glucose + 1% APEI; A + C: 1% glucose + 1% WPEI.

The growth performances of the probiotic strains are shown in Figure 1. The results showed that the use of WPEI and APEI as the only carbon sources significantly promoted the growth of the probiotic strains compared to control. Adding 1% glucose into the culture medium containing the EI polysaccharides showed a significant increase in microbial growth (*p* < 0.05), except for the *L. brevis* cultured with WPEI. Supplementation of APEI to the culture media showed a better growth effect of *L. plantarum*, *L. bulgaricus*, and *S. thermophilus* than the WPEI. The addition of 1% glucose to the culture media containing WPEI further promoted the growth of the probiotic strains.

### Food Intake and Weight Gain of Experimental Rats

**FIGURE 2:**
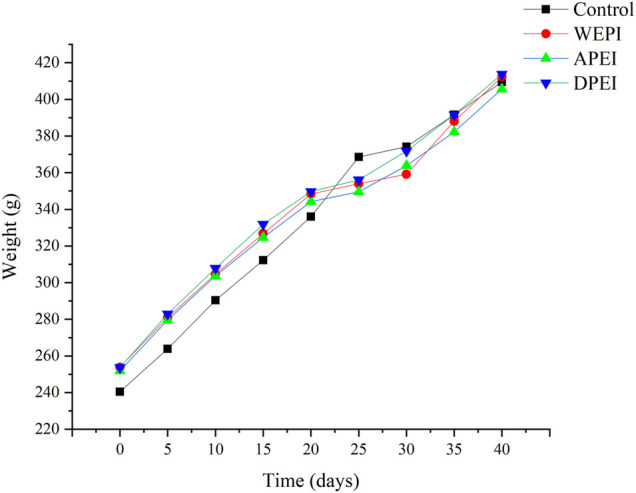
BW changes of the experimental rats.

The experimental rats were fed with rat chow (control group) and experimental diets (EI and its polysaccharide groups) for 40 days. The results showed that the control group had a higher weight gain than the other experimental groups (Figure 2; Table 4). However, no significant differences in weight gain were found between these experimental groups (*p* > 0.05). On the contrary, the rats supplemented with DPEI had a significantly higher average daily food intake than the other experimental groups. It could be due to the increased consumption of crude and dietary fibers from the DPEI increased the appetite of the rats. Therefore, the rats from the DPEI group had a lower food efficiency ratio than the other experimental and control groups.

**TABLE 4:** Food intake and BW gain of experimental rats.

Index	Control	WPEI	APEI	DPEI
Average daily weight gain (g)	6.52 ± 1.65	5.86 ± 1.14	5.64 ± 0.34	5.53 ± 1.41
Average daily food intake (g)	37.41 ± 0.23	36.27 ± 1.43	37.40 ± 2.90	45.77 ± 0.94*
Food efficiency ratio	0.17	0.16	0.15	0.12*

* *p* < 0.05.

### Changes in Visceral Organ Weights

**FIGURE 3:**
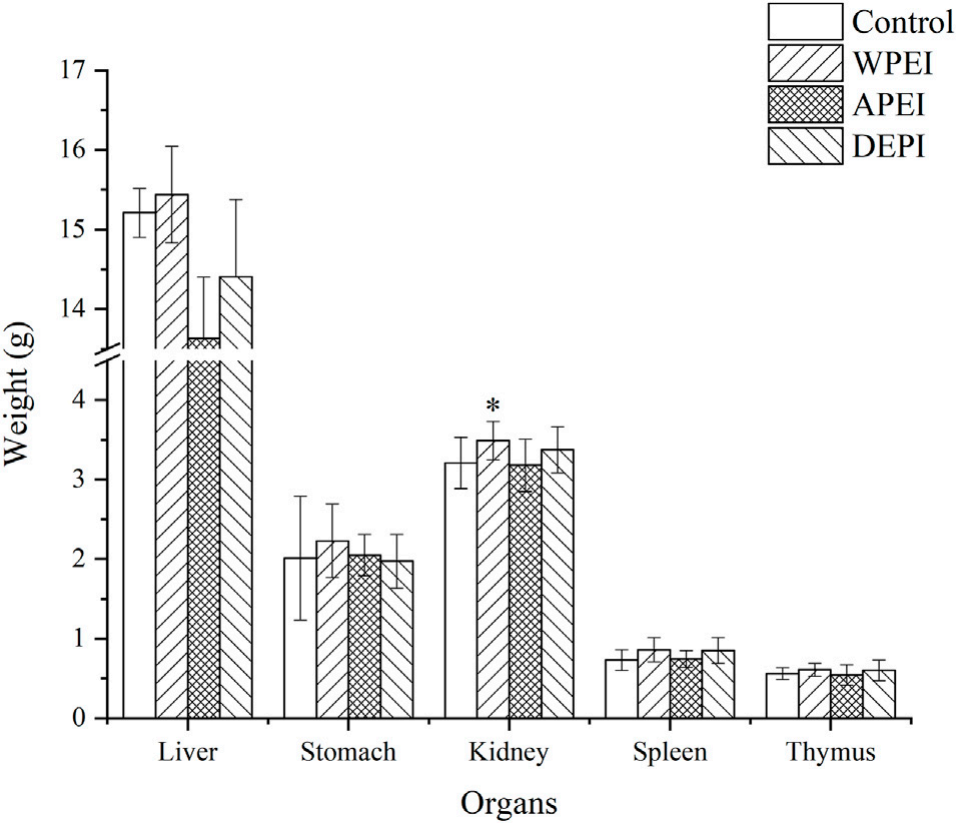
Organ weights of the experimental rats. *p < 0.05.

The liver, stomach, kidney, spleen, and thymus weights of the experimental rats are shown in Figure 3. The result showed that the average liver weight of the rats from the APEI group appeared to be lower than the other groups. The average kidney weight of the rats of the WPEI group was significantly highest (*p* < 0.05). It showed that WPEI might have caused kidney enlargement in the rats fed with 1% WPEI. Also, no significant changes in the weights of the other organs were found among the experiment groups. As shown in Table 5, the ratios of organs weights to BWs of the rats were not significantly different between the experimental groups (*p* > 0.05). The finding showed that DPEI, WPEI, and APEI were not toxic to the experimental rats.

**TABLE 5:** Ratio of organ weight to BW of thymus and spleen of experimental rats.

Group	Thymus/BW	Spleen/BW
Control	0.13 ± 0.01	0.17 ± 0.02
WPEI	0.16 ± 0.02	0.22 ± 0.04
APEI	0.14 ± 0.02	0.18 ± 0.03
DPEI	0.15 ± 0.03	0.20 ± 0.04

* *p* < 0.05; BW: body weight.

### Moisture Content and pH Values of the Feces

**FIGURE 4:**
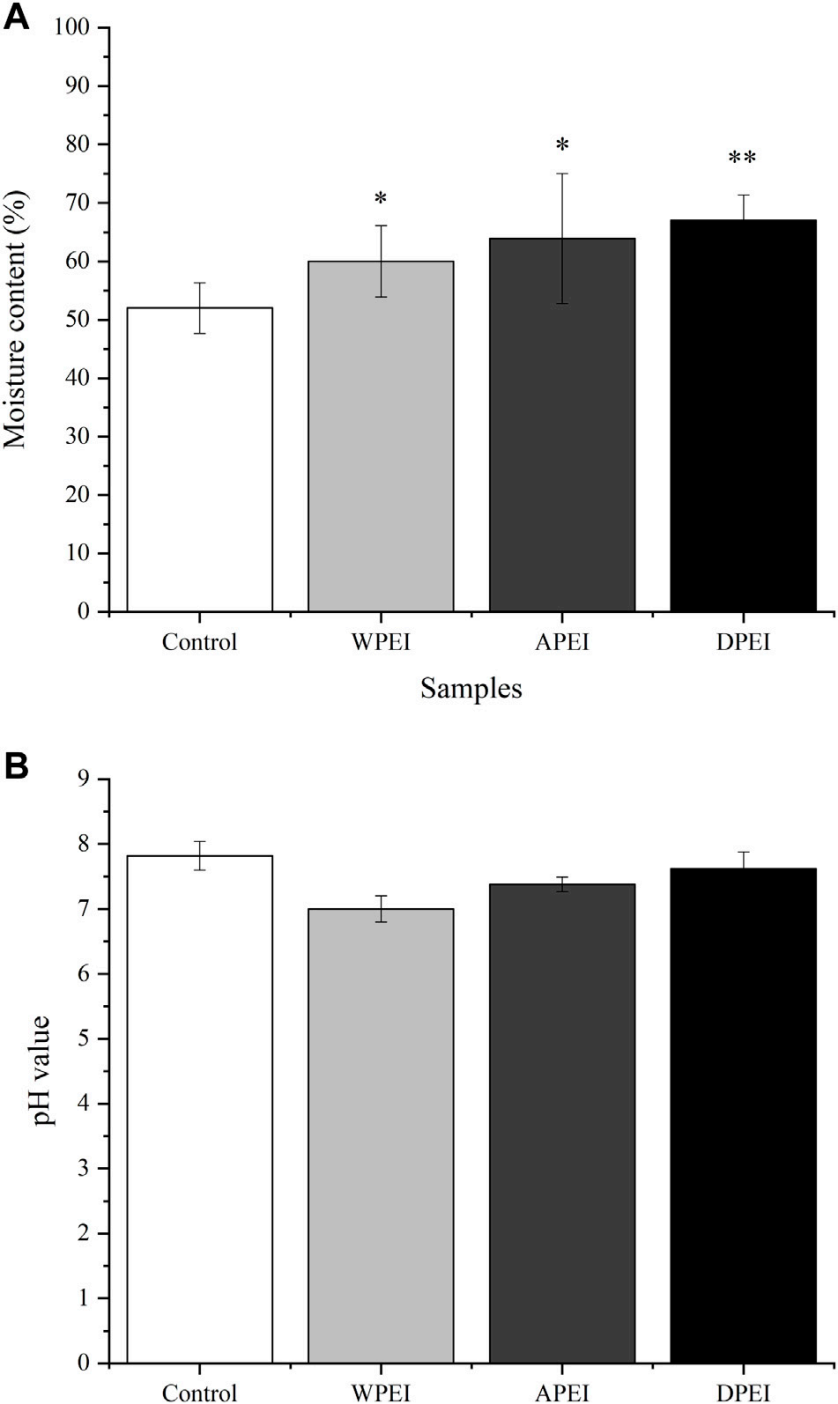
Moisture content **(A)** and pH value **(B)** of the rat feces. *p < 0.05, **p < 0.01.

As shown in Figure 4A, the moisture content of the feces collected from the rats of DPEI, WPEI, and APEI groups was significantly higher than the control group. The fecal moisture content of the DPEI group was significantly highest, where it was 67%. It shows that the crude and water-soluble fibers of EI were able to retain the water ingested. The consumption of the EI and its polysaccharides altered the pH values of the feces collected. Ingestion of WPEI has been shown to reduce the pH of feces (Figure 4B). However, no significant differences were found for the pH values of the rat feces between the experimental groups (*p* > 0.05).

### Activated Carbon Propulsion of the Experimental Rats

**FIGURE 5:**
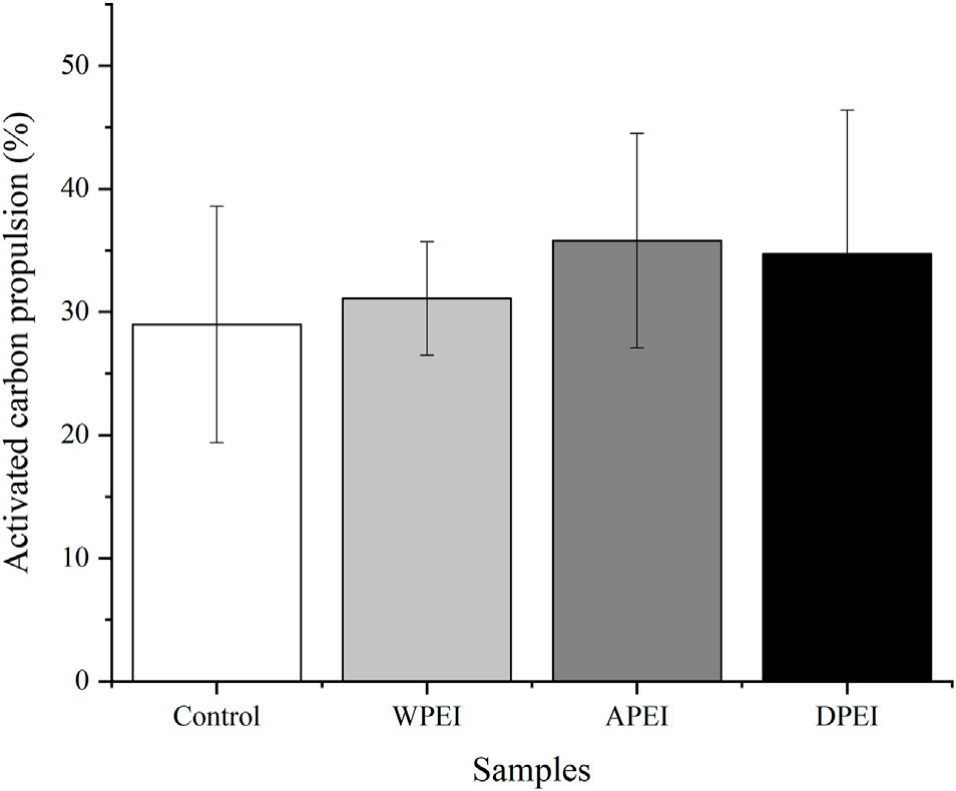
Activated carbon propulsion level of the experimental rats.

The activated carbon propulsion is linked to the movement of the small intestine. As shown in Figure 5, the activated carbon propulsion of the polysaccharide and DPEI groups was higher than the control group. However, no significant differences in the propulsion percentages were found between these groups. The level of activated carbon propulsion in the small intestine of the rats from the APEI group was 36.23%, which was higher than the WPEI and DPEI groups. The activated carbon propulsion of the control group was the lowest (29.64%). The result indicated that the EI polysaccharides could promote the peristaltic ability of the small intestine.

### SCFAs in the Feces of Experimental Rats

**FIGURE 6:**
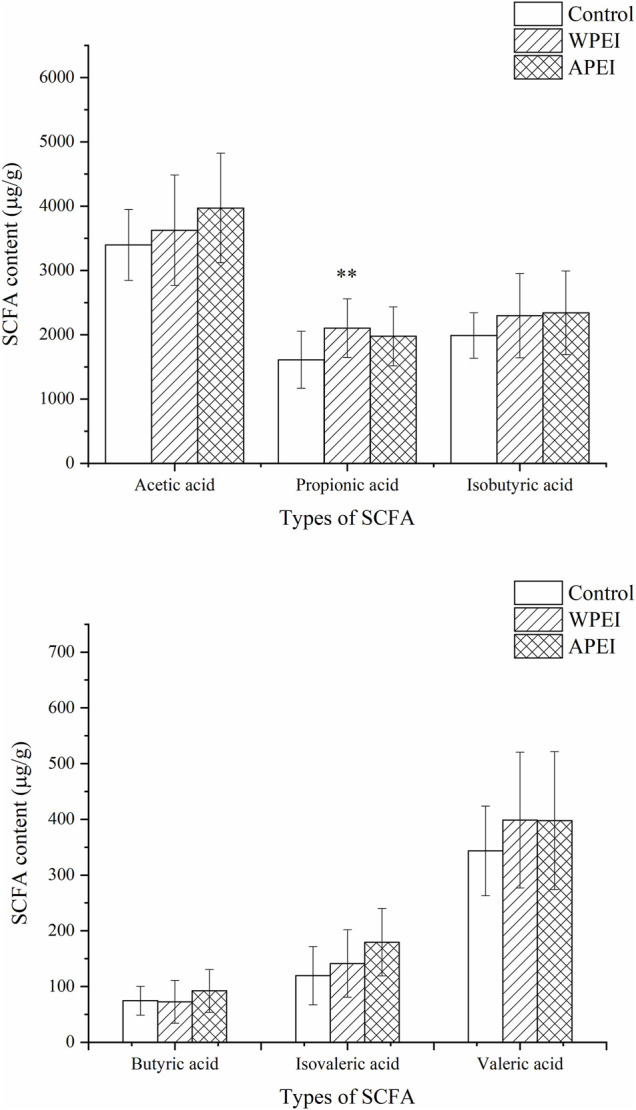
Short-chain fatty acids content of the rat feces. *p < 0.05, **p < 0.01.

In this study, the SCFAs of the rat feces were determined. SCFAs were not analyzed for the rat feces of the DPEI group because the SCFAs were not extractable from the rat feces. The SCFAs content of rat feces was not significantly different among the experimental groups (Figure 6), except for propionic acid. As shown in Figure 6, the SCFAs content of the rat feces of the polysaccharide-fed groups is higher than the control group. It showed that the SFCAs were not fully absorbed by the small intestine, especially the propionic acid. The reason is unknown.

## Discussion

In this study, the chemical compositions of EI and its polysaccharides were determined. EI is a rich source of polysaccharides. It is also a good source of minerals and some essential amino acids. The high crude and dietary fibers of EI are beneficial to the gastrointestinal tract. These fibers and polysaccharides are potent prebiotics for the intestinal microflora. Extraction of EI polysaccharides with neutral and alkaline water affects their monosaccharide compositions obtained. The alkaline extract of EI had a higher Ara, Man, and Gal, whereas the neutral water extract contained a higher amount of Rha, Xyl, and Glu. Different media pH might affect the solubility of these monosaccharides. The variation in these monosaccharides might also affect the growth-promoting effect on the growth of probiotics. Therefore, it can be developed into different types of prebiotics.

The addition of glucose to the EI polysaccharides in the growth media also further promoted microbial growth. It is because glucose is the primary energy source for cell survival (Maryanty et al., 2021). In this study, the *L. brevis* was noted to utilize glucose as the only source of energy. Besides containing a higher amount of glucose than APEI, WPEI promoted the growth of *L. brevis*, but not for the other probiotic strains. It could be because a combination of multiple forms of prebiotics in culturing intestinal microflora is better than a single source of prebiotic (Adebola et al., 2014). These data provide a new idea for the future development of prebiotics to be used in food fermentation.

EI is a polysaccharide-rich alga. It helps to reduce weight and improve blood sugar levels. In this study, the final BWs of the rats from treatment groups were lower than the control group. It might be because the EI contained crude fibers that are undigestible. The crude fibers of EI also promoted intestinal peristalsis in the rats. On top of EI fibers, the EI polysaccharides have water absorption capacity. These polysaccharides might retain water in the rat intestine. They also accelerated the intestinal peristalsis and removed harmful substances from the body through feces. The moisture content and pH of the rat feces may also reflect intestinal health. Moreover, frequent consumption of EI and its polysaccharides may ease constipation. On the contrary, literature shows that excessive fiber intake can cause side effects such as flatulence, bloating, stomach pain, and diarrhea (Makki et al., 2018). Therefore, moderate consumption of seaweed is advisable.

EI and its polysaccharides are neither toxic nor causing any harm to the human body. The polysaccharide extracts did not alter any biochemical parameters of the experimental rats. The EI extracts were also not harmful to the internal organs of the rats. It is because polysaccharides isolated from EI are natural products. These polysaccharides have a broad application prospect due to their prebiotic effects. Intestinal health can be better maintained by regular consumption of seaweed polysaccharides. These substances are foods for intestinal microflora. They can also regulate the species and proportion of intestinal microflora (Hwang et al., 2014). As seaweed contains SCFAs, it can reduce intestinal pH value and maintain intestinal homeostasis (Jiang et al., 2020). A more acidic intestinal environment hinders the growth and reproduction of pathogenic bacteria, thus reducing the risk of intestinal diseases (Ambalam et al., 2016). The polysaccharide extracts contained SCFAs, mainly acetic and valeric acids. These SCFAs provided 60–70% energy to the colon epithelial cells. It might stimulate the growth of intestinal mucosa (Donohoe et al., 2011).

SCFAs such as acetic acid, propionic acid, and butyric acid are produced by gut microbiota. Some SCFAs are sources of energy for the intestinal mucosa (Xu et al., 2021). Some of these bacteria can regulate the secretion of goblet cell mucin and mucin-related genes (Caballero-Franco et al., 2007; Dharmani et al., 2011; Sperandio et al., 2013). Regulation of the gut microbiota composition has been used as a new strategy to prevent obesity (Delzenne et al., 2011). *P. aeruginosa*, as part of the intestinal microflora, has been reported to produce butyric acid that prevents excessive production of intestinal mucus. It works with acetic acid to maintain the production of intestinal epithelium mucus (Hwang et al., 2014). Therefore, EI is a good source of prebiotics for maintaining a healthy gut.

## Conclusion

The effect of EI and its polysaccharides on intestinal function was investigated. The EI sample had a complete nutritional profile, including amino acids, polysaccharides, fibers, vitamins, and fatty acids. The probiotic strains cultured with 2% EI polysaccharides had a minor increase in log CFU/mL, especially the *L. brevis*. It showed that the polysaccharides had a mild prebiotic effect. The polysaccharides also reduced the BW of the SD rats, and DPEI had a better weight-reducing effect. The *in vivo* study also showed that WPEI and APEI were not toxic to the experimental rats. The minor increased activated carbon propulsion of APEI showed that EI polysaccharides might improve gut health. The prebiotic effect of EI and its polysaccharides was attributed to monosaccharides as a source of energy. The SCFAs of the polysaccharides could also promote the growth of intestinal mucosa. Therefore, EI and its polysaccharides can regulate intestinal health by promoting microfloral growth. It is also a potent source of prebiotics and a nutraceutical for maintaining gut health.

## Data Availability

The original contributions presented in the study are included in the article/Supplementary Material. Further inquiries can be directed to the corresponding authors.
